# Hammock: a hidden Markov model-based peptide clustering algorithm to identify protein-interaction consensus motifs in large datasets

**DOI:** 10.1093/bioinformatics/btv522

**Published:** 2015-09-05

**Authors:** Adam Krejci, Ted R. Hupp, Matej Lexa, Borivoj Vojtesek, Petr Muller

**Affiliations:** ^1^RECAMO, Masaryk Memorial Cancer Institute, Zluty kopec 7, 65653, Brno, Czech Republic,; ^2^University of Edinburgh, Institute of Genetics and Molecular Medicine, Cancer Research Centre, Edinburgh EH4 2XR, UK and; ^3^Faculty of Informatics, Masaryk University, Botanicka 68a, 60200 Brno, Czech Republic

## Abstract

**Motivation:** Proteins often recognize their interaction partners on the basis of short linear motifs located in disordered regions on proteins’ surface. Experimental techniques that study such motifs use short peptides to mimic the structural properties of interacting proteins. Continued development of these methods allows for large-scale screening, resulting in vast amounts of peptide sequences, potentially containing information on multiple protein-protein interactions. Processing of such datasets is a complex but essential task for large-scale studies investigating protein-protein interactions.

**Results:** The software tool presented in this article is able to rapidly identify multiple clusters of sequences carrying shared specificity motifs in massive datasets from various sources and generate multiple sequence alignments of identified clusters. The method was applied on a previously published smaller dataset containing distinct classes of ligands for SH3 domains, as well as on a new, an order of magnitude larger dataset containing epitopes for several monoclonal antibodies. The software successfully identified clusters of sequences mimicking epitopes of antibody targets, as well as secondary clusters revealing that the antibodies accept some deviations from original epitope sequences. Another test indicates that processing of even much larger datasets is computationally feasible.

**Availability and implementation:** Hammock is published under GNU GPL v. 3 license and is freely available as a standalone program (from http://www.recamo.cz/en/software/hammock-cluster-peptides/) or as a tool for the Galaxy toolbox (from https://toolshed.g2.bx.psu.edu/view/hammock/hammock). The source code can be downloaded from https://github.com/hammock-dev/hammock/releases.

**Contact:** muller@mou.cz

**Supplementary** **information:** Supplementary data are available at *Bioinformatics* online.

## 1 Introduction

Molecular interactions between proteins occur ubiquitously in cells and play central roles in most biological processes. These interactions are often mediated by short linear motifs located in disordered regions on the surface of one of the interacting partners (Dinkel *et* *al.*, 2013). The functional and evolutionary importance of this kind of interaction is substantial (Kim *et* *al.*, 2014). To investigate linear motif-mediated binding interactions, several experimental methods utilize short peptides to mimic structural properties of interacting proteins. Libraries containing very large numbers of such short peptide sequences can be generated easily and used to discover interaction preferences of proteins. These methods include phage display (Bratkovič, 2009) or other display-based methods, as well as technologies utilizing peptide microarrays (Halperin *et* *al.*, 2010; Legutki *et al.*, 2010; Stiffler *et al.*, 2007).

Such high-throughput methods are capable of generating huge amounts of data. The identification of true binding motifs within large datasets is a challenging task for several reasons. First, binding motifs are typically short and weak (Andreatta *et* *al.*, 2012), second, experimental origin of the data imposes the possibility of fair level of noise and most remarkably, multiple binding motifs are often contained within the data. The occurrence of more than one motif may be caused by true poly-specificity of the target, as well as by experimental imperfections. In the case of phage display, two main issues may occur. The first problematic phenomenon is nonspecific adsorption of phages to surfaces that were used to immobilize target proteins, and the second issue is caused by differences in phages’ propagation capabilities—phages may be selected on the basis of their growth capacity, rather than their binding affinity to the target (Derda *et al.*, 2011; Huang *et* *al.*, 2011).

With low-cost high-throughput methods, such as Next-Generation sequencing of phage display libraries, it is possible to obtain up to millions of unique peptide sequences (Matochko *et* *al.*, 2012). It is therefore reasonable to think of even more complicated experiments, aiming to discover multiple binding specificities of protein complexes or even whole mixtures of proteins at once. Such an experimental setup would mean an even greater number of true motifs to be identified and, generally, much more data to be processed, demanding not only better sensitivity but also adequate computational efficiency of methods employed.

Significant effort has already been put into the development of software methods for peptide data processing. Part of these tools aim to process problem-specific data, e.g. to predict binding targets of MHC molecules. These approaches utilize various techniques, including hidden Markov models (HMMs, Noguchi *et* *al.*, 2002), Gibbs sampling (Nielsen *et* *al.*, 2004) and artificial neural networks (Nielsen and Lund, 2009). It has been shown that domains interacting with short peptides are often poly-specific, which leads to correlations between residue positions of recognized motifs (Gfeller *et* *al.*, 2011). Therefore, even for a single recognition domain, it is necessary to capture these correlations, which can be done either directly, with the use of, e.g. artificial neural networks (Andreatta *et* *al.*, 2011), or indirectly, by describing one motif with correlated positions by several motifs with uncorrelated positions (Gfeller *et* *al.*, 2011). The second approach is implemented in tools using multiple position-weight matrices (also known as position-specific scoring matrices) to represent multiple specificity profiles obtained by various techniques, e.g. mixture model optimization (Kim *et* *al.*, 2011) or Gibbs sampling (Andreatta *et* *al.*, 2012). These tools try to be versatile and allow for peptide data from any biological source to be processed, but may require some prior data knowledge, such as the number of clusters to identify.

Although tools mentioned above perform well on smaller datasets of up to thousands of sequences, they have not been designed to process datasets orders of magnitude larger. In this article, we address this issue by introducing Hammock, a novel software tool for peptide sequence clustering. Hammock uses profile HMMs for precise computational representation of sequence motifs and is based on the idea of progressive cluster growth. The three key properties of this approach are (i) the ability to process very large datasets, (ii) the ability to identify multiple distinct motifs within one dataset and (iii) versatility, as no limits are put on the origin of the data, and no prior data knowledge is required.

## 2 Methods

Hammock performs several clustering steps to identify clusters of sequences sharing a motif and generate a multiple sequence alignment of each cluster. As noise often occurs, the result also contains a set of (unaligned) sequences not belonging to any cluster. Hammock utilizes HMMs to efficiently represent whole clusters of sequences at once and makes use of modern multicore processors, as all steps are parallelized.

**Fig. 1.**
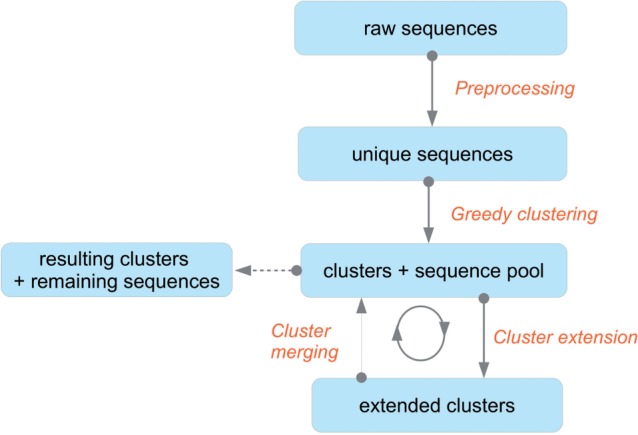
Hammock algorithm workflow. After the extraction of unique sequences, Hammock uses fast greedy clustering algorithm to identify initial cluster cores. The extension step adds more sequences into clusters and the merging step merges several clusters into one. The extension and merging steps are alternated several times with similarity requirements gradually relaxed. After the last merging step, resulting clusters and sequences not belonging to any cluster are reported

The algorithm is based on the idea of progressive cluster growth. At first, small clusters of highly similar sequences are identified, while sequences not belonging to any cluster form a set called the sequence pool. Next, two alternating steps are performed iteratively: the cluster extension step, in which sequences from the pool are inserted into clusters, and the cluster merging step, in which whole clusters are compared and merged. As the extension and merging steps are repeated, sequence-cluster and cluster-cluster similarity requirements are gradually relaxed. This leads to progressive motif discovery. At the beginning, sequence differences within a cluster are minor, and most positions are highly conserved. Later, when more sequences are added, less conserved positions emerge and sequences carrying appropriate residues on conserved positions, but possibly different residues on other positions, are allowed to join the cluster. The idea of progressive cluster growth is similar to principles used in tools aiming to identify distantly related sequences in a database, such as PSI-BLAST (Altschul, 1997) and jackhmmer from the Hmmer package (Finn *et* *al.*, 2011). The key differences between these tools and Hammock are (i) Hammock starts the iterative procedure from multiple clusters, so it performs multiple database (the sequence pool) searches in parallel; (ii) Hammock starts the iterative procedure from clusters of sequences, not from a single sequence and (iii) Hammock performs the merging step. Figure 1 shows the workflow of the algorithm.

### 2.1 Steps of the algorithm

#### 2.1.1 Pre-processing

Input data may contain multiplicities; therefore, a set of unique sequences is generated first. However, the number of times each unique sequence occurred is preserved and forms part of the final output. Moreover, Hammock supports the concept of sequence labels. Each occurrence of a sequence may optionally have a label associated, so that the information on how many times each unique sequence occurred with each label is available. The motivation for sequence labels is to offer the possibility of structuring datasets. A label may, e.g. constitute one selection or amplification round of a phage display experiment, in which phages were sequenced in several phases of the experiment.

#### 2.1.2 Initial greedy clustering

Initial clustering step identifies rather small groups of very similar sequences. To reduce computational complexity, a greedy incremental algorithm is employed. The approach is similar to algorithms used in tools for database complexity reduction (Li *et* *al.*, 2001). Sequences are first sorted by some criteria (copy number, alphabetic or random) and the first sequence becomes the representative of the first cluster. Starting from the second sequence, each sequence is compared to all current representatives. It then joins the cluster containing the most similar representative, if this similarity reaches a pre-defined threshold. Otherwise, it becomes the representative of a new cluster. To compute a similarity score, limited alignment without inner gaps and with limited maximal number of trailing gaps is performed. A substitution matrix is used to compute alignment scores.

#### 2.1.3 Cluster selection and alignment

Initial clustering results in a number of rather small clusters. Only a portion of this set is used in the next steps and the decision which clusters to use is based on their size. Remaining clusters are not treated as clusters any more and from this point on, sequences contained in them form the sequence pool.

Multiple sequence alignments of all selected clusters are generated using limited alignments produced by greedy clustering.

#### 2.1.4 Cluster extension

In this step, each cluster is represented with a profile HMM, which is then used to search the sequence pool for similar sequences and any sequences identified are added into an appropriate cluster.

Hmmer (Finn *et* *al.*, 2011) is used for both HMM construction (*hmmbuild* routine) and sequence search (*hmmsearch* routine). Local alignments are performed. Inserted sequences must be added into multiple sequence alignments. Clustal Omega (Sievers *et* *al.*, 2011) is used for this purpose. To increase resulting MSA quality, sequences are aligned to complete MSA one by one, starting from the sequence with the highest similarity score.

#### 2.1.5 Cluster merging

As the initial greedy clustering step is quite restrictive, some clusters may be very similar to each other. During the merging step, groups of similar clusters are identified and merged into one larger cluster. Local HMM-HMM alignment routine provided by HH-suite (Soding, 2004) is used to measure cluster-cluster similarity.

The cluster merging step is a bottom-up hierarchical clustering process, where clusters are progressively merged. Starting from a set of clusters *S*, clustering scheme works as follows:

First, all versus all comparisons are performed. Cluster pairs having score above a pre-defined threshold are inserted into a list *Q.* Iterative process then starts—cluster pair $\left(C_{k},C_{l}\right)$ with the highest score is removed from *Q*, along with any other pairs containing *C_k_* or *C_l_.* *C_k_* and *C_l_* are removed from *S* and merged into a new cluster *C_n_*, which is inserted into *S.* *C_n_* is compared with all the other clusters in *S* and any resulting pairs having the score above the threshold are inserted into *Q.* This process is repeated until there are no cluster pairs in *Q.*

The hierarchic clustering algorithm runs in $\Theta (N^{2})$ and guarantees optimal results by merging only the most similar cluster pair in every step. It also ensures that no cluster pair with similarity score above the threshold is left unmerged.

#### 2.1.6 Cluster merging heuristic speedup

A heuristic approach may be applied to speed the merging step up. The idea lies in pre-identification of groups of potentially similar clusters. The cluster merging algorithm is then applied only within these groups.

To identify such groups, the algorithm re-uses the information computed in the previous cluster extension step. For every cluster, a set of (distantly) similar sequences satisfying a separate, pre-defined similarity threshold is computed. If the sets of sequences distantly similar to clusters *A* and *B* have non-empty overlap, *A* and *B* are marked as *directly similar.* The transitive closure of *direct similarity* relation is called *indirect similarity*, i.e. clusters *X* and *Y* are *indirectly similar*, if there are some clusters $L_{1},L_{2}...L_{m}$ such that *X* is *directly similar* to *L*_1_, *L*_1_ is *directly similar* to *L*_2_ etc. and *L_m_* is *directly similar* to *Y.* A group of potentially similar clusters is then defined as such group where every cluster is *indirectly similar* to each other.

The cluster merging algorithm is performed within each group of potentially similar clusters. Although resulting time complexity stays the same, time requirements are typically reduced for two reasons: First, some groups of potentially similar clusters may contain one cluster only, for which no comparisons will be performed. Second, as the merging routine runs in quadratic time, the computation benefits from the division into subgroups. On the other hand, this approach no longer guarantees optimal results and may lead to less clusters being merged, compared with the full cluster merging routine.

#### 2.1.7 Iterating the extension and merging steps

The extension and merging steps are repeated (three times by default). As the heuristic merging speedup may lead to less clusters being merged, complete cluster merging procedure is performed in the last round, when there are less clusters and therefore time requirements are reduced.

### 2.2 Cluster diversity control

In every step, HMM match states are by default defined as alignment columns having less than 5% gaps and minimal information content of 1.2. For clusters not to become too diverse, a minimal number of HMM match states (4 by default) is maintained. The total number of positions and the number of inner gaps in clusters’ multiple sequence alignments are also limited. If two clusters are about to be merged or a sequence is about to be inserted into a cluster, but the resulting cluster would not satisfy these constraints, the insertion or merging is not performed. These checks assure that no cluster can become overly diverse in any step.

### 2.3 Implementation

Hammock is implemented on the Java platform. External programs (Clustal Omega, Hmmer, HH-suite) are compiled separately and called from within the Java code as external processes.

### 2.4 Galaxy implementation

To offer a GUI and server functionality, a XML wrapper was created to allow Hammock to be used as a tool in the Galaxy toolbox (Giardine, 2005; Goecks *et* *al.*, 2010). The wrapper also ensures easy installation by automatically downloading all external components from online sources.

## 3 Results

Hammock was used to process two datasets of different sizes and complexities. Another collection of large, both real and simulated datasets was utilized to investigate computation time requirements. To maintain consistency, we present results obtained by runs with default parameters. To visualize multiple sequence alignments of resulting clusters, we use sequence logos generated by WebLogo 3.4 (Crooks, 2004) throughout the article. Only alignment columns containing less than 50% gaps are shown.

### 3.1 Human SH3 domain

This rather small dataset comprises 2457 sequences from a phage display experiment, and it was previously used in two studies aiming for the development of peptide clustering and multiple specificity identification tools (Andreatta *et* *al.*, 2012; Kim *et* *al.*, 2011). It contains sequences binding to Src SH3 domain, which is known to possess binding specificities of both class I SH3 domains (motif [R/K]xxPxxP) and class II SH3 domains (motif PxxPx[R/K]). Apart from sequences carrying these motifs, the dataset also contains noise.

**Fig. 2.**
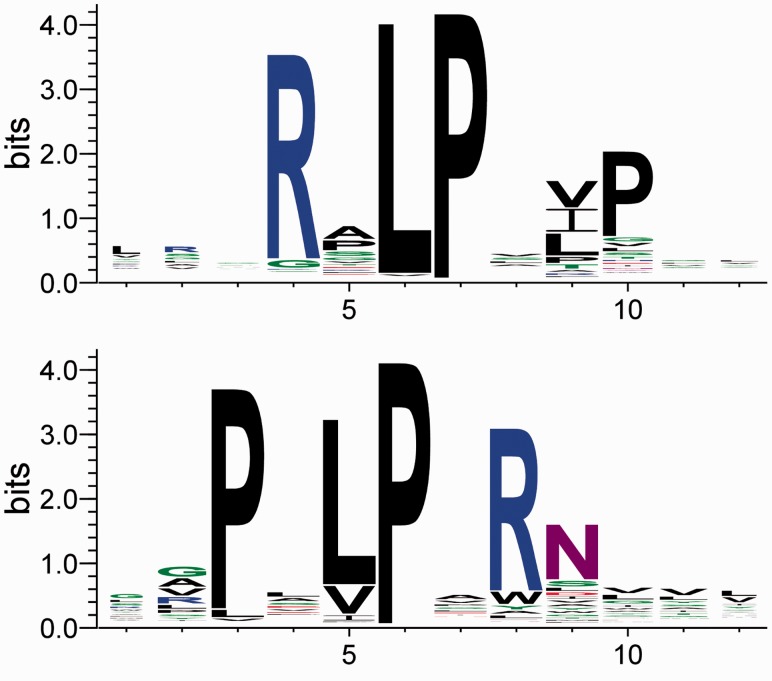
Sequence logos of resulting clusters carrying the SH3 domain binding motifs. Cluster carrying class I motif (top) contains 1738 sequences, and cluster carrying class II motif (bottom) contains 415 sequences

Hammock successfully identified two clusters, each consisting of sequences carrying a binding motif corresponding to canonical motif of one SH3 domain class. With default parameters, these are the only clusters reported in final results. Class I cluster contains 1738 sequences, class II cluster 415 sequences and 304 sequences were not assigned to any cluster. Sequence logos of these clusters are shown in Figure 2.

### 3.2 Monoclonal antibodies

To investigate the tool’s performance on a more complex dataset, a phage display experiment examining three monocolonal antibodies (CHIP 3.1, EEV1‐2.1 and DO.1) was performed. Three rounds of selection and two rounds of amplification were performed, and phages were sequenced after each round of selection or amplification using Illumina HiSeq instrument. In total, 74 041 unique sequences were obtained. The total sequence copy number was 389 873. Sequences were divided into 15 groups (three selection and two amplification groups for each of three antibodies). See Supplementary Section S2 for experimental design details.

The motivation for sequencing after each round of selection or amplification lies in the effort to detect experimental artifacts and understand the whole process of the phage display experiment. It was shown that some phages may evince nonspecific binding to surfaces used for immobilization of target molecules, while others may possess exceptional ability to amplify in bacteria. These two categories of phages are then present in the data, regardless of their actual binding affinity to target molecules. In terms of sums of sequence copy numbers, clusters consisting of sequences binding non-specifically should be significantly increased in size in selection rounds for all antibodies. Clusters housing sequences of phages with exceptional amplification ability should be increased in size in all amplification rounds. On the contrary, cluster diversity (i.e. the number of unique sequences within a cluster) is expected to decrease gradually, as each round of both selection and amplification eliminates several non-binding or non-amplifying clones.

**Fig. 3.**
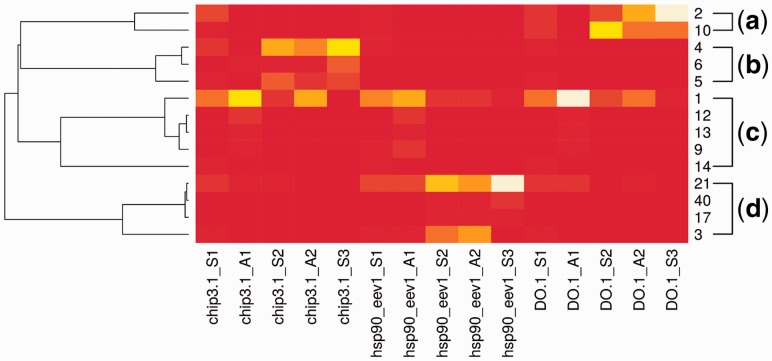
A heatmap of sequence occurrences in all categories for 14 of the largest clusters. Each row represents a category profile corresponding to one cluster (cluster ids are listed on the right side) and each column represents one category. There are 15 categories—three antibodies, for each antibody three selection rounds (S1, S2 and S3) and two amplification rounds (A1, A2). The heatmap is normalized for category. A row dendrogram is generated on the basis of row correlation [the distance between rows *v*_1_ and *v*_2_ is $1-\text{cor}(v_{1},v_{2})$], where cor is the Pearson correlation coefficient). Four major groups of category profiles [(a), (b), (c) and (d)] are highlighted

The dataset was clustered using Hammock with default parameters, which resulted in 74 clusters, together containing 14 421 (19.5% of the dataset) unique sequences with the copy number sum of 316 119 (81.1% of the dataset). There are 14 clusters containing each at least 1% of the dataset’s copy number sum and these together contain 81.9% of all sequences contained in clusters. A heatmap visualizing the sums of copy numbers of sequences in each category for each of 14 largest clusters is shown in Figure 3. For detailed information on all 74 clusters, see Supplementary Table S1.

**Fig. 4.**
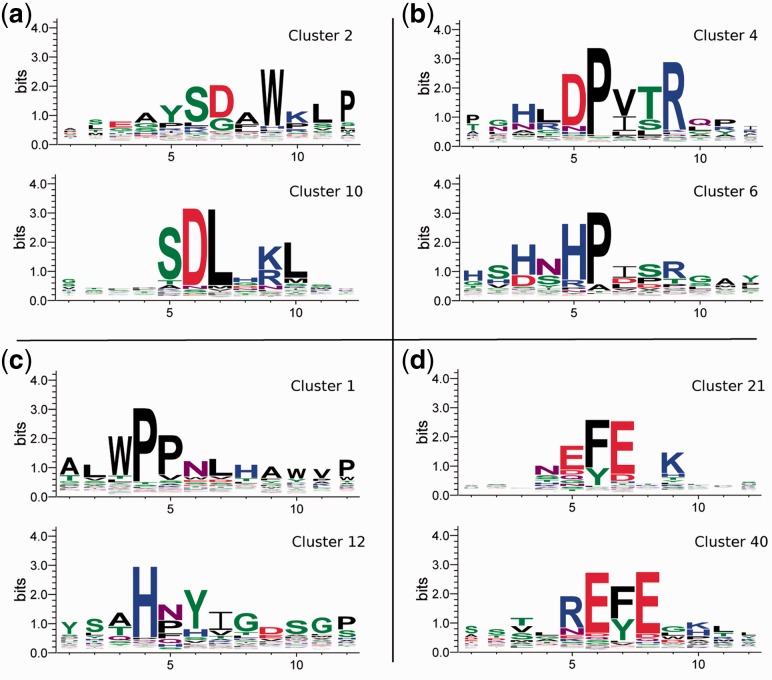
Sequence logos of clusters from four groups of category profiles. For each group, the largest cluster and the cluster closest to it (in terms of category profile correlation) are displayed. Based on expected behavior, group (**a**) corresponds to sequences binding to DO.1, group (**b**) to sequences binding to CHIP 3.1, group (**c**) to sequences with exceptional ability to multiply and group (**d**) to sequences binding to EEV1‐2.1

The heatmap shows four major groups of cluster category profiles. Three groups evince the behavior expected for true binders to a single antibody—majority of sequences occur in categories corresponding to a single antibody, and relative cluster sizes are significantly increased in selection rounds. Each of these groups contains one large cluster and several smaller secondary clusters. According to the heatmap, cluster 4 should contain sequences binding to CHIP 3.1, cluster 21 binders to EEV1‐2.1 and cluster 2 binders to DO.1. On the contrary, the fourth group containing large cluster 1 and four smaller clusters evince the behavior expected for sequences with exceptional ability to multiply—large amounts of sequences appear in categories corresponding to all antibodies and cluster size is significantly increased in amplification rounds. Sequence logos of the major cluster and one secondary cluster (the closest one according to row correlation) for each of four groups are shown in Figure 4.

Sequence logos of clusters 2, 4 and 21 show strong sequence similarity to regions of actual targets of examined antibodies (listed in Table 1). This fact confirms that these clusters contain sequences actually binding the antibodies and so that the motifs correspond to antibody epitopes. In the case of DO.1, independent studies are available, confirming that cluster 2 carries the actual sequence of the epitope (Stephen *et al.*, 1995; Vojtesek *et* *al.*, 1992). Moreover, secondary clusters 10, 6 and 40 possess similar motifs, which means that these clusters contain sequences that mimic the same epitope. Sequence differences between the main and secondary clusters suggest that the antibodies are able to tolerate small deviations from the epitope sequences of their actual targets. Clusters having category profiles with lower correlation to main clusters 2, 4 and 21 also evince lower levels of sequence similarity. See Supplementary Table S2 for complete matrix of correlations and Supplementary Figure S1 for complete list of sequence logos of the 14 largest clusters.

**Table 1.** Epitopes corresponding to cluster sequence logos

Group	Antibody	Target protein	Epitope location	Epitope seq.
(a)	DO-1	p53	20–25	SDLWKL
(b)	CHIP 3.1	CHIP	265–273	GHFDPVTRS
(d)	EEV1‐2.1	HSP90-alpha	535–541	KEFEGKT

Locations and sequences of putative epitopes for each antibody are listed. The locations are stated in amino acid counts from the N-terminus. Sequence logos of corresponding clusters carry strong sequence similarity to these regions of the original antibody targets.

The sequence logo of cluster 1 shows no significant sequence similarity to the target of any antibody. It mainly consists of a single sequence ALWPPNLHAWVP, which occurs in 56 758 copies and therefore occupies 92% of cluster 1. It constitutes 14.5% of the whole dataset, and it is the most popular sequence of all, which confirms that this sequence has exceptional ability to multiply. Clusters having a high correlation of category profiles to the profile of cluster 1 possess no significant sequence similarity to cluster 1. This fact suggests that exceptional ability to multiply is not based on one sequence motif and even completely different sequences may evince this kind of behavior.

The diversity of the clusters (i.e. the number of unique sequences within a cluster) is the highest in the first selection rounds. For a majority of clusters, the differences between the numbers of unique sequences in the first selection rounds between different antibodies are minor, which indicates that the efficiency of the first round of selection was rather low. Cluster diversity generally decreases in later selection rounds, as expected. Amplification rounds always cause a big drop in cluster diversity, while in some cases, the diversity is slightly increased by the subsequent selection round. This observation is in agreement with the assumption of significant differences in phage propagation capabilities. After the amplification round, several over-amplified clones fill most of the sequencing capacity, while less popular clones become undetectable at given sequencing depth. The subsequent selection round substantially decreases the number of copies of over-amplified clones and thus allows for the detection of less frequent clones, if still present. See Supplementary Figure S2 for the heatmap of diversity of the 14 largest clusters.

The sequence composition of almost all of the clusters follows the power law pattern - there are a few sequences with very high copy number and many sequences with low copy number. This pattern is also evident within the dataset as a whole. This finding is in agreement with previous findings of Derda *et* *al.* (2011) and Matochko *et* *al.* (2012), who state that the distribution of sequence copy numbers in phage display experiments is far from linear.

The category profiles of sequences within some clusters are not totally homogenous, which suggests that such clusters contain some noise. For example, in some cases, a sequence evincing the behavior expected for phages with exceptional ability to multiply is contained within a cluster the overall category profile of which falls into the category of true binders. This suggests that the sequence similarity of such sequence to the rest of the cluster is rather random and is not connected with any similarity in binding preferences. As these cases are quite rare, such low level of noise does not significantly affect the overall properties of the identified clusters. If more noise of this kind appears in some dataset, we suggest the user to use stricter parameters for cluster merging and extension.

### 3.3 Comparison with existing tools

To compare Hammock with the two tools mentioned before (MUSI and the Gibbs sampling tool), both the datasets were processed by all of the tools on the same high-end desktop computer (AMD-FX 9370 8-core CPU, 4.4 GHz, 32GB RAM, Linux Mint 17).

As the measure of clustering quality, we use the Kullback–Leibler divergence (KLD) as defined in Andreatta *et* *al.* (2012). We state KLD calculated over both match states as defined in Hammock and over all MSA positions. See Supplementary Section S1 for precise definition and formulas used.

For the SH3 dataset, the results of all runs were fairly consistent. All the tools identified the two clusters representing class I and class II domain motifs, sequence logos of corresponding clusters are very similar. Hammock removed the most sequences from dataset (304), while achieving the highest KLD.

For the antibodies dataset, the Hammock parameters were left at default values (which means to search for up to 250 clusters), MUSI was set to search for up to 100 clusters (−m 100 option) and the Gibbs sampling tool was run three times, set to use the trash cluster and to start from 2, 10 and 100 clusters, respectively.

Here, the differences in run times and results were substantial. While Hammock finishes in under 3 min, both the other tools need hours to finish, with the Gibbs sampling tool only being able to finish within 72 h when starting from 100 clusters. The differences in the quality of clustering results were also large. MUSI failed to report any useful information, as it puts all the 74 041 sequences in one extremely diverse cluster. The Gibbs sampling tool reported 100 clusters, but the overall KLD of this system was low compared with the result reported by Hammock, which was therefore superior in both the quality of the results and run time.

Hammock removes more sequences from the result than the other tools. While this is not so significant for the (relatively clean) SH3 dataset, in case of the (very noisy) antibodies dataset, the difference is huge. Therefore, it is reasonable to think of using Hammock as a de-noising tool, i.e. to process a dataset with Hammock and use the results as an input for another tool. This approach was tested on both datasets. In case of the SH3 dataset, pre-filtering improves the quality of the results of both the tools, but neither of them achieves the KLD of Hammock alone. For the antibodies dataset, MUSI still fails to provide meaningful results and places all the sequences into one cluster, but the quality of the results obtained by the Gibbs sampling tool is competitive to Hammock and when the number of clusters reported by Hammock (74) is used as initial number of clusters for the Gibbs sampling tool, the KLD of the result is even slightly higher. This suggests that in some cases, Hammock may be used to remove noise and estimate optimal number of clusters prior to the use of some other, resource-heavy algorithm. The results of all the runs are summarized in Table 2.

**Table 2.** A summary of the results obtained by running Hammock, MUSI and the Gibbs sampling tool on the SH3 and the antibodies datasets

Dataset	Sequences	Tool	Params	Time	No. of clusters	No. of sequences	KLD match	KLD all
SH3	2457	Hammock	-t 8	17 s	2	2153	28.165	24.858
SH3	2457	Gibbs	-trash	39 min 3 s	2	2450	25.151	22.369
SH3	2457	MUSI		17 s	2	2456	20.96	19.89
Antibodies	74 041	Hammock	-t 8	2 min 35 s	74	14 421	17.897	17.635
Antibodies	74 041	Gibbs	-trash	>72 h	—	—	—	—
Antibodies	74 041	Gibbs	-trash -g 10	>72 h	—	—	—	—
Antibodies	74 041	Gibbs	-trash -g 100	14 h 13 min 53 s	100	74 040	12.622	11.335
Antibodies	74 041	MUSI	-m 100	8 h 20 min 1 s	1	74 041	0.0	3.22
SH3 filtered	2153	Gibbs		27 min 36 s	2	2153	26.865	23.94
SH3 filtered	2153	MUSI		25 s	2	2152	23.57	23.25
Antibodies filtered	14 421	Gibbs		>72 h	—	—	—	—
Antibodies filtered	14 421	Gibbs	-g 10	4 h 19 min 27 s	10	14 421	10.47	12.215
Antibodies filtered	14 421	Gibbs	-g 100	21 min 6 s	89	14 421	18.863	17.427
Antibodies filtered	14 421	Gibbs	-g 74	30 min 45 s	68	14 421	18.78	17.81
Antibodies filtered	14 421	MUSI	-m 100	2 min 25 s	1	14 421	0	2.97

Runs of MUSI and the Gibbs sampling tool on these two datasets pre-filtered by Hammock are also stated. Columns are (from the left): dataset name, the number of unique sequences in the dataset, tool name, additional tool parameters, run time, number of clusters in the result, number of unique sequences in the result, KLD calculated over MSA columns defined as match stated by Hammock and KLD calculated over all MSA columns.

### 3.4 Performance testing

Two large peptide datasets were used to test Hammock’s time requirements. Both come from phage display experiments and contain sequences 12 amino acids long. The first is a pseudorandom dataset generated from the monoclonal antibodies dataset mentioned earlier. It contains both original sequences and sequences in which random amino acid substitutions were introduced, with respect to overall amino acid frequencies. The second dataset comes from the work of Matochko *et* *al.* (2012).

Random subsets of various sizes up to 10^6^ unique sequences were sampled from both datasets and processed by Hammock. The same desktop computer as in previous section was used. All parameters were left at default values except for cluster core selection after greedy clustering. Five percent of largest clusters were selected for further clustering in every dataset (the default is 2.5% with the maximum of 250 clusters).

**Fig. 5.**
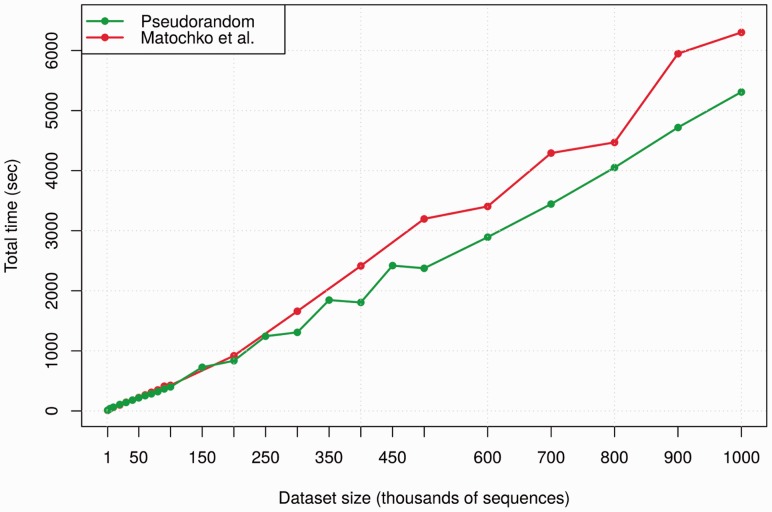
Run times of random subsets of two test datasets. Subsets of up to 10^6^ unique sequences were randomly selected from one dataset containing real phage display data and one pseudorandom dataset. See Supplementary Table S3 for numeric values of data points displayed in this figure

Run times are shown in Figure 5. Times are fairly data dependent, run times for pseudorandom dataset are shorter. We assume that this is caused by the lack of real motifs and therefore lower complexity of this dataset.

## 4 Discussion

We presented Hammock—a software tool for short peptide sequence clustering. The tool is able to cluster large amounts of data containing noise and to produce multiple sequence alignments of resulting clusters. The main motivation to create Hammock was to provide the ability of processing datasets originating from large-scale screening of combinatorial peptide libraries, such as phage display. Nevertheless, the tool is universal and no limits are put on the origin or format of processed data (Hammock accepts input in three formats including fasta) and no prior data knowledge, such as the number of clusters to identify, is needed. Therefore, Hammock is applicable for a range of other data sources, such as variable regions of lymphocyte receptors or viral proteins. It accepts virtually any set of peptide sequences as input.

We performed a pilot large-scale phage display experiment which shows the way future experiments based on combinatorial peptide libraries could take. We demonstrated that Hammock is capable of processing data originating from such an experiment and provide valuable biological insights, even when the data contain noise. We also demonstrated Hammock’s time requirements on datasets of various sizes.

Compared with existing tools, Hammock is much faster and can process datasets orders of magnitude larger, while it achieves the best quality of clustering results. Another difference is that with default parameters, Hammock may remove many sequences from the result, if they do not fit any cluster well. This feature is beneficial in the case of large and noisy datasets and can be utilized by using Hammock as a de-noising tool. On the other hand, in the case of small and clean datasets containing subtle sequence motifs only, this behavior may not be desired. It can be changed by parameter tuning, but we estimate that for such cases, some of more computationally intensive methods, such as Gibbs sampling, may be more suitable.

There are a number of parameters allowing the user to influence clustering results. In this article, Hammock was shown to perform well with default parameters, but parameter tuning will often be desirable to accommodate the diverse nature of input data and the diverse spectrum of biological questions that require answering. If a strong motif and less noise is present in the dataset, or coarse-grained clustering is desired, cluster merging and extension thresholds should be lowered. If a large number of small and specialized clusters is needed, thresholds should be increased.

Hammock comes as a standalone program, as well as a tool for the Galaxy toolbox. Galaxy provides a GUI, server functionalities and also an online storage called the Galaxy tool shed (Blankenberg *et* *al.*, 2014), which allows any Galaxy server administrator or local instance user to install Hammock with all its dependencies just by virtually one click. The Galaxy implementation makes Hammock more user-friendly and allows for broad community of non-expert users to use it easily.

In conclusion, Hammock satisfies the need for extremely large peptide datasets to be processed and thus allows for novel types of experiments based on data from various sources to be performed. The user-friendly Galaxy implementation gives the possibility to use this tool to a broad spectrum of potential users.

## Supplementary Material

Supplementary Data
