# Supplementary material for: Hammock: a hidden Markov model-based peptide clustering algorithm to identify protein-interaction consensus motifs in large datasets
Source: Bioinformatics. 2015 Sep 5;32(1):9–16. doi: 10.1093/bioinformatics/btv522 (PMC4681989; doi:10.1093/bioinformatics/btv522)
Supplement: Supplementary Data [file supp_32_1_9__index.html]

Hammock: a hidden Markov model-based peptide clustering algorithm to identify protein-interaction consensus motifs in large datasets — Hammock: a hidden Markov model-based peptide clustering algorithm to identify protein-interaction consensus motifs in large datasets — Supplementary Data 

# Hammock: a hidden Markov model-based peptide clustering algorithm to identify protein-interaction consensus motifs in large datasets

## Supplementary Data

files

- Supplementary Data - pdf file
