# Supplementary material for: Hammock: a hidden Markov model-based peptide clustering algorithm to identify protein-interaction consensus motifs in large datasets
Source: Bioinformatics. 2015 Sep 5;32(1):9–16. doi: 10.1093/bioinformatics/btv522 (PMC4681989; doi:10.1093/bioinformatics/btv522)
Supplement: Supplementary Data [file supp_btv522_supplementary.pdf]

# Hammock: A Hidden Markov model-based peptide clustering to identify protein-interaction consensus motifs in large datasets

## Supplementary Information

Adam Krejci, Theorodre Hupp, Matej Lexa, Borivoj Vojtesek, and Petr Muller

## S.1 KullbackLeibler divergence calculation

The KullbackLeibler divergence (KLD) is calculated in the way described in Andreatta *et al.* (2012), i.e. using Log-odds (LO) matrices adjusted for small samples.

### LO matrix calculation

For each alignment column $j$, the value of LO matrix for amino acid $A$ is calculated as

$$LO_{A,j} = \frac{n}{n+\sigma} \log \frac{p'_{A,j}}{q_A}$$

where $p'_{A,j}$ is the pseudo-count corrected frequency of amino acid $A$ at position $j$, $q_A$ is the background frequency of amino acid $A$, $n$ is the number of sequences in the alignment and $\sigma$ is a weight factor adjusting LO matrix values for very small clusters. As in Andreatta *et al.* (2012), $\sigma$ is set to 10.

The background frequencies $q_A$ are defined as follows (values stated are frequencies times $10^3$):

| A | R | N | D | C | Q | E | G | H | I | L | K | M | F | P | S | T | W | Y | V |
|---|---|---|---|---|---|---|---|---|---|---|---|---|---|---|---|---|---|---|---|
| 74 | 52 | 45 | 54 | 25 | 34 | 54 | 74 | 26 | 68 | 99 | 58 | 25 | 47 | 39 | 57 | 51 | 13 | 32 | 73 |

The pseudocount correction is performed as described in Nielsen *et al.* (2004) and Altschul (1997). No sequence weighting is performed. The effective amino-acid frequency $p'_{A,j}$ is calculated as

$$p'_{A,j} = \frac{n \cdot p_{A,j} + \beta \cdot g_{A,j}}{n+\beta}$$

where $n$ is the number of sequences in the alignment, $p_{A,j}$ is the observed frequency of amino acid $A$ in column $j$, $\beta$ is the weight on pseudocounts (set to 200, according to Andreatta *et al.* (2012) and $g_{A,j}$ is the pseudocount frequency of amino acid $A$ in column $j$ calculated as

$$g_{A,j} = \sum_m p_{m,j} \cdot q_{A,m}$$

where $p_{m,j}$ is the observed frequency of each amino acid $m$ and $q_{A,m}$ is the target frequency implicit in the (row-normalized) substitution matrix, in this case, Blosum 62 (Henikoff and Henikoff, 1992).

### Scoring

The score $S(x, LO)$ of a peptide $x$ aligned to a LO matrix is calculated as the sum of LO values for amino acids from $x$ at match state positions:

$$S(x, LO) = \sum_j LO_{X,j}$$

where $j$ is the index over match state positions and $X, j$ is the amino acid from $x$ at position $j$.

To evaluate the fitness of a peptide $x$ in a cluster $C$ (where $x$ is part of the cluster's multiple alignment), $S_x$ score is calculated, but peptide $x$ is excluded from the LO matrix used. The fitness of a cluster $C$ is then the average fitness of all its peptides:

$$KLD_C = \frac{\sum_l S(c_l, LO'_l)}{n}$$

where $l$ is the index over peptides in the cluster, $c_l$ is l-th peptide from cluster $C$, $LO'_l$ is a LO matrix of the alignment without peptide $c_l$ and $n$ is the number of sequences in the cluster.

Finally, the KLD of a whole system of clusters $K$ is calculated as the average fitness of all the peptides in the system.

$$KLD_K = \frac{\sum_k \sum_l S(c_{k,l}, LO'_{k,l})}{\sum_k n_k}$$

where $k$ is the index over clusters in the system.

Scale factor for matrix used is $\frac{2}{\log 2}$, to be consistent with Andreatta *et al.* (2012).

## S.2 The antibodies phage display experiment

### Antibody preparation

We used mouse monoclonal antibody DO-1 recognizing human p53 protein (uniprot P04637), EEV1-2.1 recognizing human Hsp90 (uniprot P07900 and P08238) and CHIP3.1 recognizing human protein CHIP (uniprot Q9UNE7). The antibodies were dissolved in Phosphate Buffered Saline (PBS) at concentration 1 mg/ml before binding to solid matrices. 20 µl per sample of suspended Protein G Sepharose 4 FF (GE Healthcare) was used to immobilize the antibodies in the first round selection, 1.5 mg of Dynabeads protein A (Life technologies) was used to immobilize the antibodies in the second round and 1.5 mg of Dynabeads protein A (Life technologies) was used to immobilize the antibodies in third round of selection. The bead sediments were resuspended in 100 µl of antibody solution (1 mg/ml) and incubated for 30 min on a rotary shaker at room temperature. The excess unbound antibody was washed by 3 times wash in 1 ml of panning buffer containing bovine serum albumin 10 mg/ml, 50 mM TRIS pH 7.5 and 0.5 % Tween20. The beads were finaly resuspended in 100 µl of panning buffer.

### Phage display

The Ph.D.™-12 Phage Display Peptide Library (New Englang Biolabs) was used in all presented experiments. We used 10 µl of original library containing $10^{11}$ pfu to select the interacting peptides in the first round. The library was panned in a buffer containing bovine serum albumin 10 mg/ml,

50 mM TRIS pH 7.5 and 0.5 % Tween20. The phage library was incubated for 1 hour with antibody-coated beads on a rotary shaker at 4 °C. Unbound phages were removed by 3 washes in the panning buffer and 1 last wash in PBS. Captured phages were eluted by suspending the bead pellets in 100 µl of an elution buffer containing 0.1 M Glycine pH 3.0. The supernatant containing eluted phages was separated into new tubes and was neutralized by the addition of 10 µl of 1 M Tris pH 8.5. This solution of pahges was subsequently used for tittering, amplification, and sequencing. The amplification and tittering of the library was performed according to the original protocol for Ph.D.™-12 Phage Display Peptide Library.

### Amplification and sequencing of phage DNA

The sequencing library was prepared by the amplification of a 124 bp region of the phage DNA containing the variable 36 bp sequence. Herculase II fusion polymerase (Agilent) was used to amplify the sequence with forward primer `GTGACTGGAGTTCAGACGTGTGCTCTTCCGATCTNNNcgcaattcctttagtggtacc` and reverse primer `ACACGACGCTCTTCCGATCTctgtatgggattttgctaac`, where small letters indicate phage-specific sequence and underlined part stays for the internal index. The PCR reaction of 50 µl contained 10 µl of crude eluted phage or 100 ng of DNA isolated from amplified phage, 300 nM primers, 500 mM Betain, 1x reaction buffer and 1 U of Herculase II enzyme. The PCR reaction was run under these conditions: 95 °C - 60 s; (95 °C - 15 s; 55 °C - 30 s)×30. The purified PCR amplicons were sequenced on the Illumina HiSeq platform at Otogenetics Corporation (Norcross, Georgia, US).

The resulting FASTQ files were filtered on quality and a custom Java application was used to extract the 36 bp regions of interest along with appropriate internal index sequences. The extracted regions were further filtered to satisfy the codon constraints specific for the Ph.D.™-12 Phage Display Peptide Library, translated and formatted into the FASTA format.

## S.3 How to reproduce the results, a quick guide

### Prerequisities

Java, version at least 1.7.0 is required.
Suppose Hammock was downloaded and extracted to `~/Hammock/`
On Linux-based 64 bit systems, compilation of external tools should not be necessary. On other systems, please download and compile appropriate versions of all tools (Clustal Omega v. 1.2.0, HH-suite v. 2.0.16, Hmmer v. 3.1b1) and move binaries to appropriate locations in Hammock folder (for Clustal Omega: file `Hammock/clustal-omega-1.2.0/clustalO-64bit`, for HH-suite, complete structure of tools' folder in `Hammock/hhsuite-2.0.16`, for Hmmer, files `Hammock/hmmer-3.1b1/src/hmmbuild` and `Hammock/hmmer-3.1b1/src/hmmsearch`). Alternatively, paths to files or commands for installed tools can be changed in `Hammock/settings/settings.prop` file.

### To process the SH3 (MUSI) dataset

```
export HHLIB=~/Hammock/hhsuite-2.0.16/lib/hh/
java -jar ~/Hammock/dist/Hammock.jar full -i ~/Hammock/examples/musi.fa
```

This will create a folder `Hammock/dist/Hammock_result_1` containing the results. Output folder can be changed using parameter -o. By default, 4 threads are used. This number can be changed using parameter -t.

**To process the antibodies dataset**

```
export HHLIB=~/Hammock/hhsuite-2.0.16/lib/hh/
java -jar ~/Hammock/dist/Hammock.jar full -i ~/Hammock/examples/antibodies.fa
```

This will create a folder `Hammock/dist/Hammock_result_1` (or `Hammock_result_2`, if `Hammock_result_1` already exists) containing the results. Output folder can be changed using parameter -o. By default, 4 threads are used. This number can be changed using parameter -t. By default, label columns in resulting tables are sorted according to sum of sequences with each label. Default ordering of labels can be manually changed using parameter -l, for example:

```
export HHLIB=~/Hammock/hhsuite-2.0.16/lib/hh/
java -jar ~/Hammock/dist/Hammock.jar full -i ~/Hammock/examples/antibodies.fa -l
chip3.1_S1,chip3.1_A1,chip3.1_S2,chip3.1_A2,chip3.1_S3,hsp90_eev1_S1,hsp90_eev1_A1
,hsp90_eev1_S2,hsp90_eev1_A2,hsp90_eev1_S3,DO-1_S1,DO-1_A1,DO-1_S2,DO-1_A2,DO-1_S3
```

For more details, see the full manual at `http://www.recamo.cz/userfiles/file/Software/Hammock/Hammock-manual.pdf`

| cluster_id | main_sequence | sum | chip3.1_S1 | chip3.1_A1 | chip3.1_S2 | chip3.1_A2 | chip3.1_S3 | hsp90_eev1_S1 | hsp90_eev1_A1 | hsp90_eev1_S2 | hsp90_eev1_A2 | hsp90_eev1_S3 | DO-1_S1 | DO-1_A1 | DO-1_S2 | DO-1_A2 | DO-1_S3 |
|---|---|---|---|---|---|---|---|---|---|---|---|---|---|---|---|---|---|
| 1 | ALWPPNLHAWVP | 61700 | 665 | 12707 | 1286 | 8775 | 957 | 1369 | 6205 | 1476 | 1551 | 790 | 1286 | 13733 | 3359 | 6495 | 1046 |
| 21 | AHSANNFDVKGI | 33640 | 368 | 1080 | 545 | 697 | 459 | 743 | 2340 | 6290 | 4802 | 11002 | 608 | 2258 | 804 | 1290 | 354 |
| 2 | EAYSDAWKLPNT | 33114 | 412 | 692 | 450 | 560 | 375 | 363 | 362 | 373 | 323 | 464 | 679 | 527 | 3625 | 10310 | 13599 |
| 4 | NLDPITRQYITP | 30944 | 321 | 584 | 3613 | 7213 | 15299 | 326 | 490 | 352 | 315 | 358 | 370 | 434 | 525 | 456 | 288 |
| 10 | GSFPSDLNRLMR | 28015 | 236 | 507 | 456 | 557 | 574 | 280 | 369 | 269 | 271 | 268 | 488 | 462 | 11769 | 5977 | 5532 |
| 3 | ESVQLPKWMEYE | 12796 | 146 | 299 | 259 | 202 | 150 | 252 | 1341 | 4048 | 4669 | 229 | 255 | 199 | 316 | 253 | 178 |
| 6 | HSHNHPISRGAY | 11558 | 98 | 1114 | 802 | 2050 | 5690 | 168 | 212 | 120 | 104 | 142 | 204 | 323 | 228 | 192 | 111 |
| 5 | HVAPSWWTARLF | 10444 | 194 | 171 | 2025 | 2774 | 3745 | 127 | 135 | 117 | 99 | 124 | 320 | 172 | 204 | 142 | 95 |
| 9 | MPLMSEPALEML | 8243 | 111 | 1825 | 185 | 593 | 125 | 314 | 1564 | 269 | 209 | 158 | 228 | 1643 | 361 | 565 | 93 |
| 12 | YSAHNYIGDSGP | 7579 | 90 | 2141 | 184 | 810 | 120 | 184 | 1446 | 197 | 192 | 114 | 179 | 1009 | 295 | 521 | 97 |
| 13 | TPMVERNYNAAD | 6915 | 72 | 1718 | 160 | 634 | 106 | 158 | 1325 | 218 | 191 | 106 | 144 | 1154 | 305 | 546 | 78 |
| 17 | LRASEWEWKTQR | 5074 | 79 | 105 | 143 | 89 | 80 | 153 | 358 | 947 | 708 | 1414 | 145 | 298 | 254 | 203 | 98 |
| 40 | SVLREYELHTLH | 4511 | 56 | 64 | 71 | 57 | 56 | 96 | 135 | 1116 | 726 | 1787 | 86 | 73 | 78 | 65 | 45 |
| 14 | NELPYRLWHWPR | 4383 | 247 | 338 | 308 | 332 | 268 | 293 | 269 | 271 | 251 | 289 | 303 | 297 | 413 | 303 | 201 |
| 16 | SSYNFGDEFHMK | 3544 | 39 | 53 | 55 | 54 | 56 | 67 | 60 | 530 | 321 | 2053 | 43 | 49 | 67 | 52 | 45 |
| 19 | TLSDLAQHMMSS | 2940 | 26 | 121 | 50 | 84 | 46 | 64 | 155 | 72 | 67 | 325 | 73 | 53 | 1163 | 370 | 271 |
| 18 | HWPGWLERGSPA | 2802 | 128 | 187 | 187 | 194 | 196 | 128 | 327 | 137 | 133 | 152 | 185 | 264 | 248 | 227 | 109 |
| 22 | RLDASSMISGRV | 2604 | 162 | 408 | 96 | 126 | 37 | 120 | 394 | 67 | 67 | 29 | 108 | 564 | 148 | 250 | 28 |
| 122 | IPIANPEFEMLV | 2492 | 32 | 41 | 47 | 33 | 31 | 66 | 116 | 718 | 453 | 744 | 49 | 47 | 44 | 44 | 27 |
| 37 | GSASPWAHEFET | 2378 | 33 | 78 | 265 | 431 | 48 | 63 | 80 | 463 | 366 | 146 | 67 | 78 | 135 | 72 | 53 |
| 25 | NEYHYHRSQLQV | 2077 | 24 | 40 | 51 | 19 | 28 | 54 | 68 | 714 | 614 | 172 | 73 | 42 | 88 | 49 | 41 |
| 42 | TGAPPRLDARPA | 2061 | 434 | 34 | 171 | 188 | 183 | 136 | 35 | 26 | 22 | 21 | 196 | 25 | 309 | 186 | 95 |
| 53 | APPLKEPTSSSI | 2038 | 21 | 165 | 60 | 65 | 18 | 93 | 424 | 77 | 72 | 27 | 95 | 266 | 386 | 188 | 81 |
| 39 | TDHNHNLREFEF | 1942 | 127 | 37 | 44 | 34 | 21 | 44 | 46 | 242 | 155 | 859 | 181 | 42 | 53 | 33 | 24 |
| 29 | YDHSATHLVSLP | 1763 | 46 | 663 | 79 | 163 | 30 | 68 | 124 | 61 | 44 | 33 | 168 | 136 | 70 | 61 | 17 |
| 50 | VDVDETNNQSYP | 1501 | 18 | 404 | 55 | 124 | 18 | 66 | 278 | 63 | 22 | 19 | 55 | 212 | 76 | 71 | 20 |
| 34 | ESHPTLLHWTQA | 1470 | 29 | 357 | 116 | 181 | 94 | 55 | 110 | 41 | 29 | 36 | 73 | 164 | 87 | 75 | 23 |
| 57 | TGTSWESGKVLK | 1394 | 24 | 35 | 39 | 22 | 24 | 48 | 44 | 322 | 178 | 311 | 48 | 153 | 66 | 60 | 20 |
| 35 | GQTVMMRTFGER | 1387 | 11 | 307 | 38 | 154 | 19 | 45 | 468 | 71 | 60 | 28 | 43 | 43 | 43 | 41 | 16 |
| 33 | DDHTFTDAPKLL | 1350 | 15 | 26 | 26 | 18 | 16 | 14 | 12 | 15 | 13 | 14 | 40 | 17 | 529 | 285 | 310 |
| 38 | SGVYKVAYDWQH | 1281 | 190 | 28 | 123 | 30 | 26 | 95 | 22 | 31 | 22 | 45 | 500 | 50 | 68 | 29 | 22 |
| 98 | NHYLSYPSNPGI | 1199 | 28 | 60 | 64 | 32 | 19 | 72 | 207 | 124 | 90 | 148 | 68 | 120 | 84 | 70 | 13 |
| 45 | FPWKSNWVEFEM | 1063 | 28 | 15 | 39 | 18 | 13 | 25 | 35 | 193 | 70 | 475 | 83 | 17 | 24 | 17 | 11 |
| 48 | HLTHSPIPVRAM | 1016 | 26 | 244 | 41 | 88 | 13 | 19 | 81 | 27 | 15 | 16 | 62 | 190 | 87 | 79 | 28 |
| 62 | SAINTATALHIR | 996 | 13 | 417 | 47 | 133 | 17 | 30 | 41 | 110 | 59 | 28 | 41 | 16 | 16 | 15 | 13 |
| 93 | VIGTPDSSTHGL | 891 | 19 | 167 | 59 | 57 | 16 | 81 | 96 | 28 | 28 | 21 | 73 | 118 | 64 | 47 | 17 |
| 74 | HVVKQAMSNNMM | 868 | 13 | 88 | 46 | 46 | 12 | 19 | 215 | 26 | 30 | 8 | 31 | 77 | 121 | 88 | 48 |
| 58 | YFDWGLSNANDT | 845 | 13 | 241 | 35 | 103 | 13 | 29 | 136 | 25 | 23 | 10 | 38 | 84 | 43 | 38 | 14 |
| 92 | SYSALDHPPHPT | 826 | 21 | 28 | 44 | 13 | 22 | 38 | 185 | 29 | 17 | 11 | 69 | 51 | 171 | 114 | 13 |
| 85 | GLDLNMSTWMLN | 801 | 22 | 263 | 43 | 35 | 10 | 63 | 57 | 17 | 12 | 18 | 45 | 57 | 86 | 53 | 20 |
| 54 | DVFRAATIRTAS | 724 | 224 | 10 | 43 | 18 | 13 | 28 | 13 | 13 | 14 | 9 | 267 | 22 | 27 | 10 | 13 |
| 109 | THLMTEIRWPWY | 703 | 26 | 19 | 42 | 3 | 9 | 22 | 35 | 129 | 55 | 8 | 38 | 21 | 205 | 65 | 26 |
| 116 | SQDIRTWNGTRS | 700 | 50 | 57 | 73 | 25 | 12 | 63 | 45 | 29 | 17 | 31 | 154 | 57 | 53 | 25 | 9 |
| 123 | ADQTHTRNLLRQ | 698 | 122 | 38 | 23 | 20 | 9 | 75 | 78 | 83 | 50 | 32 | 81 | 36 | 27 | 18 | 6 |
| 60 | TNVDQHHTVDWL | 680 | 14 | 392 | 37 | 92 | 13 | 23 | 14 | 9 | 4 | 9 | 27 | 15 | 16 | 12 | 3 |
| 104 | TYTTKPWEEFEV | 657 | 12 | 11 | 32 | 5 | 12 | 23 | 22 | 155 | 80 | 225 | 31 | 14 | 11 | 14 | 10 |
| 143 | YLVPRTPAQHPG | 643 | 21 | 9 | 34 | 20 | 21 | 37 | 15 | 13 | 10 | 11 | 50 | 15 | 217 | 90 | 80 |
| 84 | GATANVYTTQWG | 630 | 10 | 166 | 31 | 62 | 17 | 26 | 70 | 21 | 6 | 5 | 34 | 100 | 44 | 31 | 7 |
| 94 | SWSYDPRQIMMT | 595 | 19 | 19 | 127 | 165 | 45 | 19 | 70 | 20 | 11 | 9 | 25 | 7 | 38 | 17 | 4 |
| 68 | IECCRLVTNTTP | 587 | 10 | 45 | 13 | 21 | 8 | 27 | 115 | 32 | 13 | 5 | 17 | 157 | 47 | 73 | 4 |
| 71 | NPVSMEYGGKQS | 547 | 6 | 7 | 6 | 9 | 9 | 15 | 19 | 222 | 194 | 18 | 14 | 6 | 11 | 2 | 9 |
| 111 | DESPYYVPRTPA | 521 | 7 | 9 | 22 | 7 | 7 | 16 | 16 | 17 | 17 | 39 | 24 | 20 | 149 | 82 | 89 |
| 106 | LDRQLEWMYLSR | 515 | 35 | 31 | 41 | 36 | 32 | 32 | 25 | 62 | 38 | 21 | 27 | 33 | 46 | 35 | 21 |
| 81 | IGSPHQTAPLLT | 512 | 11 | 44 | 19 | 12 | 6 | 35 | 85 | 16 | 21 | 11 | 32 | 87 | 39 | 86 | 8 |
| 110 | EREYDMQKLISY | 506 | 59 | 12 | 14 | 12 | 9 | 14 | 11 | 49 | 41 | 160 | 89 | 11 | 12 | 9 | 4 |
| 86 | SLPILFMPQHYS | 480 | 6 | 212 | 20 | 78 | 7 | 16 | 21 | 6 | 7 | 10 | 27 | 24 | 28 | 14 | 4 |
| 119 | ANYHPWSLMSDK | 366 | 15 | 113 | 33 | 55 | 13 | 15 | 10 | 5 | 2 | 8 | 37 | 25 | 17 | 13 | 5 |
| 161 | NPMLLSMGQKPA | 356 | 13 | 12 | 20 | 11 | 9 | 21 | 65 | 17 | 14 | 4 | 31 | 70 | 38 | 29 | 2 |
| 180 | TLDMHGSSPRQL | 334 | 9 | 11 | 24 | 6 | 3 | 14 | 150 | 25 | 18 | 6 | 32 | 7 | 19 | 5 | 5 |
| 105 | VHLQAGELMNPK | 333 | 5 | 78 | 12 | 33 | 7 | 13 | 45 | 3 | 9 | 0 | 10 | 59 | 26 | 24 | 9 |
| 158 | YTCGAVSTAQPC | 332 | 81 | 13 | 22 | 8 | 5 | 34 | 19 | 4 | 3 | 5 | 46 | 52 | 19 | 18 | 3 |
| 138 | FPIDWYVSTRAT | 273 | 9 | 3 | 16 | 3 | 4 | 13 | 18 | 4 | 5 | 3 | 15 | 4 | 123 | 46 | 7 |
| 140 | DLFGSRHNPGNI | 264 | 93 | 4 | 30 | 4 | 5 | 47 | 8 | 5 | 0 | 7 | 44 | 4 | 7 | 3 | 3 |
| 232 | MPITTYWLSQYR | 261 | 16 | 37 | 38 | 13 | 4 | 21 | 20 | 6 | 7 | 7 | 31 | 26 | 22 | 6 | 7 |
| 149 | STLSPTDYMRHT | 260 | 9 | 56 | 23 | 18 | 3 | 10 | 4 | 8 | 7 | 9 | 22 | 41 | 16 | 30 | 4 |
| 200 | LPSPAGPHDSFE | 195 | 10 | 3 | 17 | 6 | 4 | 7 | 7 | 49 | 34 | 28 | 12 | 4 | 8 | 3 | 3 |
| 214 | AWNNTTPHFESK | 186 | 6 | 2 | 12 | 8 | 6 | 15 | 6 | 64 | 23 | 12 | 10 | 7 | 7 | 6 | 2 |
| 194 | EKQQFDTLKQNP | 130 | 1 | 2 | 6 | 3 | 3 | 5 | 3 | 42 | 17 | 39 | 3 | 0 | 0 | 2 | 4 |
| 253 | GLTMVQGLKEFE | 126 | 1 | 0 | 4 | 2 | 5 | 1 | 5 | 43 | 12 | 39 | 5 | 3 | 2 | 2 | 2 |
| 250 | SALKGLFPADHH | 125 | 13 | 27 | 15 | 6 | 2 | 3 | 4 | 2 | 2 | 4 | 26 | 4 | 11 | 4 | 2 |
| 216 | TQGMCARSPNCT | 122 | 1 | 5 | 17 | 3 | 0 | 11 | 4 | 5 | 0 | 0 | 15 | 27 | 19 | 14 | 1 |
| 376 | QHYWNEYEIPFQ | 112 | 6 | 3 | 8 | 0 | 2 | 10 | 3 | 23 | 13 | 19 | 9 | 1 | 11 | 1 | 3 |
| 293 | WPGLSRAHHGTV | 105 | 21 | 6 | 6 | 1 | 3 | 8 | 32 | 2 | 2 | 1 | 18 | 0 | 3 | 1 | 1 |
| 279 | DRWVARDPASIF | 96 | 15 | 3 | 10 | 1 | 3 | 7 | 1 | 1 | 2 | 2 | 36 | 0 | 8 | 6 | 1 |

Table S1: **Overview of clustering results for the antibodies dataset**. Each line represents one resulting cluster. The column `main_sequence` contains the most abundant sequence in each cluster. The total size of each cluster and amounts of sequences in each category are stated. The table is sorted from the largest clusters to the smallest.

| | 1 | 2 | 3 | 4 | 5 | 6 | 7 | 8 | 9 | 10 | 11 | 12 | 13 | 14 |
|---|---|---|---|---|---|---|---|---|---|---|---|---|---|---|
| 1 | 0 | 0.419 | 1.284 | 1.306 | 1.264 | 1.272 | 1.313 | 1.320 | 1.325 | 1.089 | 1.329 | 1.288 | 1.326 | 1.263 |
| 2 | 0.419 | 0 | 1.259 | 1.278 | 1.248 | 1.230 | 1.296 | 1.287 | 1.299 | 1.083 | 1.345 | 1.307 | 1.311 | 1.265 |
| 3 | 1.284 | 1.259 | 0 | 0.052 | 0.111 | 1.262 | 1.255 | 1.296 | 1.327 | 1.056 | 1.350 | 1.293 | 1.365 | 1.262 |
| 4 | 1.306 | 1.278 | 0.052 | 0 | 0.255 | 1.153 | 1.124 | 1.175 | 1.203 | 1.068 | 1.372 | 1.322 | 1.399 | 1.293 |
| 5 | 1.264 | 1.248 | 0.111 | 0.255 | 0 | 1.217 | 1.216 | 1.267 | 1.281 | 0.842 | 1.351 | 1.292 | 1.322 | 1.262 |
| 6 | 1.272 | 1.230 | 1.262 | 1.153 | 1.217 | 0 | 0.183 | 0.147 | 0.154 | 0.991 | 1.330 | 1.429 | 1.371 | 1.255 |
| 7 | 1.313 | 1.296 | 1.255 | 1.124 | 1.216 | 0.183 | 0 | 0.015 | 0.072 | 0.941 | 1.233 | 1.331 | 1.258 | 1.133 |
| 8 | 1.320 | 1.287 | 1.296 | 1.175 | 1.267 | 0.147 | 0.015 | 0 | 0.045 | 0.994 | 1.213 | 1.326 | 1.244 | 1.112 |
| 9 | 1.325 | 1.299 | 1.327 | 1.203 | 1.281 | 0.154 | 0.072 | 0.045 | 0 | 0.798 | 1.179 | 1.297 | 1.171 | 1.119 |
| 10 | 1.089 | 1.083 | 1.056 | 1.068 | 0.842 | 0.991 | 0.941 | 0.994 | 0.798 | 0 | 1.017 | 1.000 | 0.857 | 1.017 |
| 11 | 1.329 | 1.345 | 1.350 | 1.372 | 1.351 | 1.330 | 1.233 | 1.213 | 1.179 | 1.017 | 0 | 0.013 | 0.020 | 0.513 |
| 12 | 1.288 | 1.307 | 1.293 | 1.322 | 1.292 | 1.429 | 1.331 | 1.326 | 1.297 | 1.000 | 0.013 | 0 | 0.025 | 0.506 |
| 13 | 1.326 | 1.311 | 1.365 | 1.399 | 1.322 | 1.371 | 1.258 | 1.244 | 1.171 | 0.857 | 0.020 | 0.025 | 0 | 0.457 |
| 14 | 1.263 | 1.265 | 1.262 | 1.293 | 1.262 | 1.255 | 1.133 | 1.112 | 1.119 | 1.017 | 0.513 | 0.506 | 0.457 | 0 |

Table S2: **Table of correlation-based distance between 14 largest clusters from the antibodies dataset**. Normalized amounts of sequences within each of 15 categories form a vector of length 15 for each cluster. The distance between vectors $v_1$ and $v_2$ is then computed as $1-cor(v_1, v_2))$ where $cor$ is the Pearson correlation coefficient.

| unique sequences | time pseudorandom (s) | time matochko (s) |
|---|---|---|
| 1000 | 10.592 | 9.35 |
| 2000 | 18.046 | 17.139 |
| 5000 | 38.666 | 36.451 |
| 10000 | 62.945 | 60.648 |
| 20000 | 106.41 | 100.417 |
| 30000 | 141.639 | 144.286 |
| 40000 | 180.092 | 179.805 |
| 50000 | 215.931 | 222.831 |
| 60000 | 253.4 | 265.263 |
| 70000 | 283.461 | 309.756 |
| 80000 | 321.459 | 349.715 |
| 90000 | 366.695 | 411.717 |
| 100000 | 399.213 | 426.257 |
| 150000 | 727.446 | NA |
| 200000 | 834.747 | 918.438 |
| 250000 | 1243.978 | NA |
| 300000 | 1308.411 | 1660.36 |
| 350000 | 1845.977 | NA |
| 400000 | 1805.957 | 2414.153 |
| 450000 | 2419.692 | NA |
| 500000 | 2375.185 | 3197.307 |
| 600000 | 2892.586 | 3404.279 |
| 700000 | 3444.12 | 4293.217 |
| 800000 | 4050.89 | 4470.755 |
| 900000 | 4719.001 | 5948.539 |
| 1000000 | 5310.038 | 6301.207 |

Table S3: **Table of run times displayed in Fig. 5 in the article.** Time values are stated in seconds.

| order | # of clusters | # of sequences | # of cl1 sequences | KLD match | KLD all |
|---|---|---|---|---|---|
| size | 74 | 14421 | 753 | 17.897 | 17.635 |
| alphabetic | 71 | 12294 | 661 | 18.065 | 18.1 |
| random 42 | 70 | 12898 | 626 | 18.709 | 18.656 |
| random 198 | 63 | 12742 | 902 | 17.86 | 17.52 |
| random 9827 | 73 | 12899 | 631 | 18.6 | 17.96 |

Table S4: **Comparison of sorting strategies for the greedy clustering step**. The antibodies dataset was clustered using Hammock with default parameters and different sorting strategies. For random sorting, 3 different seeds of the pseudorandom number generator were used. Column *# of cl1 sequences* states the number of unique sequences in the largest cluster containing the most frequent sequence ALWPPNLHAWVP.

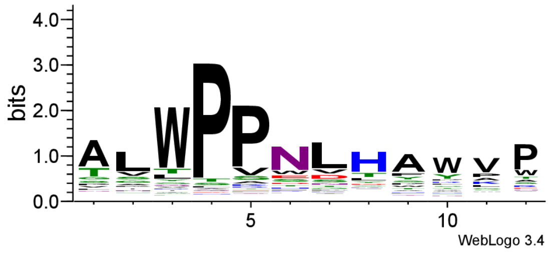


(a) cluster 1

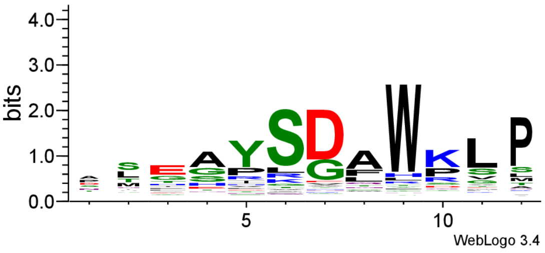


(b) cluster 2

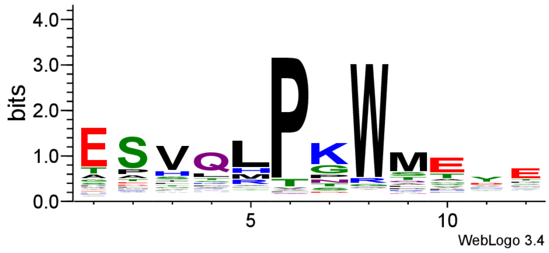


(c) cluster 3

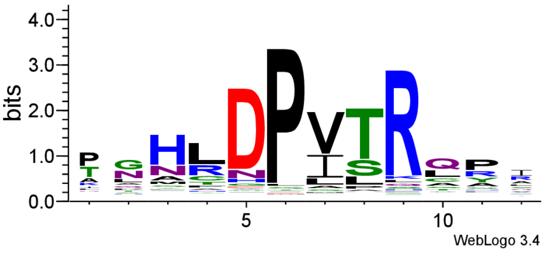


(d) cluster 4

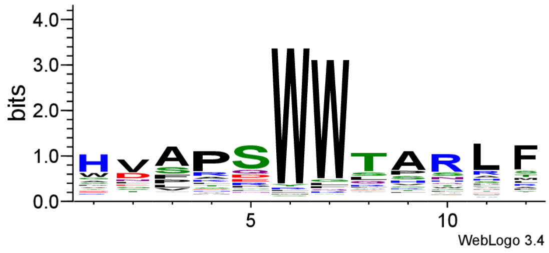


(e) cluster 5

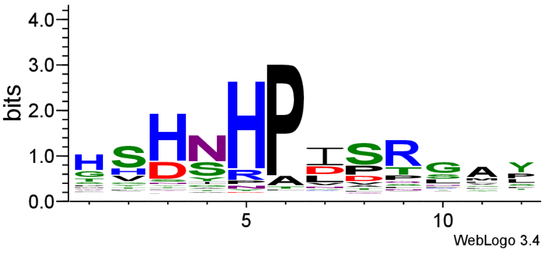


(f) cluster 6

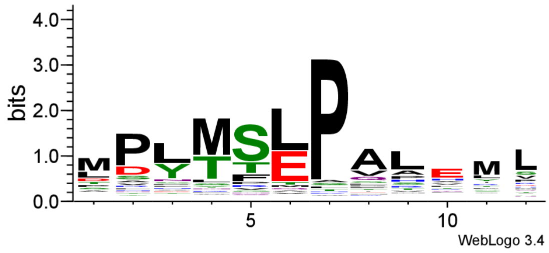


(g) cluster 9

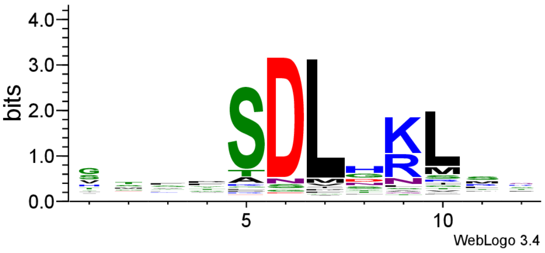


(h) cluster 10

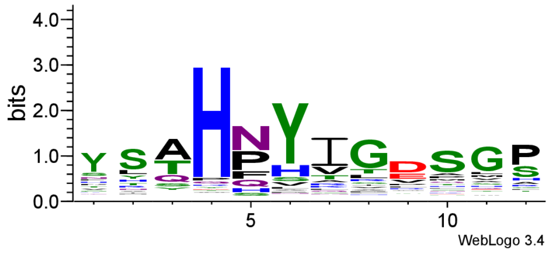


(i) cluster 12

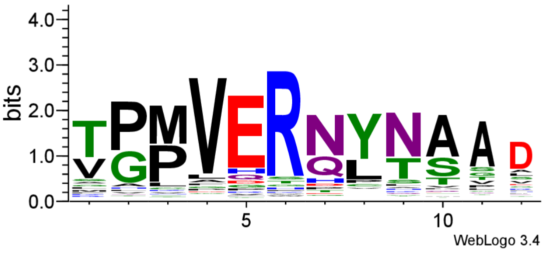


(j) cluster 13

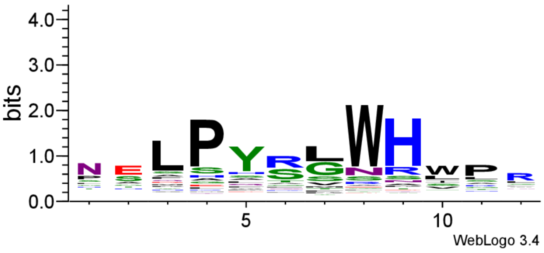


(a) cluster 14

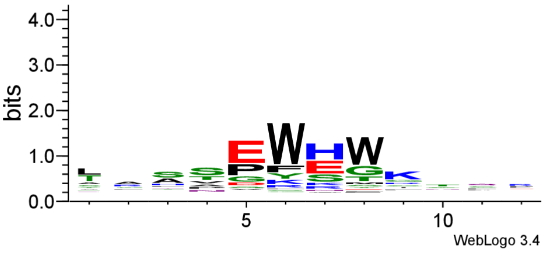


(b) cluster 17

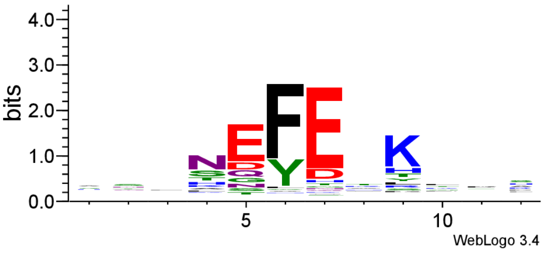


(c) cluster 21

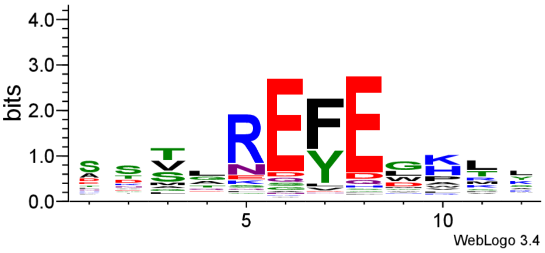


(d) cluster 40

Figure S1: **Sequence logos of all 14 largest clusters from the antibodies dataset.**

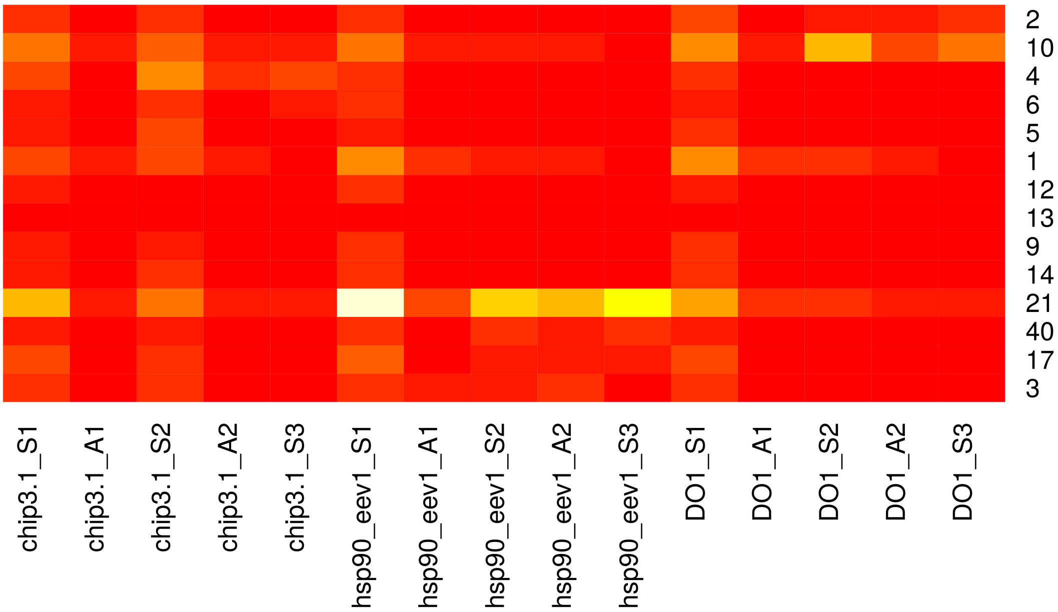


Figure S2: **A heatmap of diversity (=unique sequence counts) in all categories for 14 of the largest clusters.** Each row represents a category profile corresponding to one cluster (cluster ids are listed on the right side) and each column represents one category. There are 15 categories—3 antibodies, for each antibody 3 selection rounds (S1, S2, S3) and 2 amplification rounds (A1, A2). The heatmap is normalized for total read count in each category.

# References

Altschul, S. (1997). Gapped BLAST and PSI-BLAST: a new generation of protein database search programs. *Nucleic Acids Research*, **25**(17), 3389–3402.

Andreatta, M., Lund, O., and Nielsen, M. (2012). Simultaneous alignment and clustering of peptide data using a gibbs sampling approach. *Bioinformatics*, **29**(1), 8–14.

Henikoff, S. and Henikoff, J. G. (1992). Amino acid substitution matrices from protein blocks. *Proceedings of the National Academy of Sciences*, **89**(22), 10915–10919.

Nielsen, M., Lundegaard, C., Worning, P., Hvid, C. S., Lamberth, K., Buus, S., Brunak, S., and Lund, O. (2004). Improved prediction of MHC class i and class II epitopes using a novel gibbs sampling approach. *Bioinformatics*, **20**(9), 1388–1397.
